# Supplementary material for: Increased Susceptibility of WHIM Mice to Papillomavirus-induced Disease is Dependent upon Immune Cell Dysfunction
Source: PLoS Pathog. 2024 Sep 3;20(9):e1012472. doi: 10.1371/journal.ppat.1012472 (PMC11398641; doi:10.1371/journal.ppat.1012472)
Supplement: S1 Fig — Female reproductive tracts of WHIM mice (WT/M) and wildtype littermates (WT/WT) were infected with 5x10^6 VGE MmuPV1. Lavage samples were collected at 2 weeks and 6 weeks post infection. Blue line indicated MmuPV1 copy number in lavage samples from historically mock-infected control mice (background level of MmuPV1 DNA qPCR). Each line represents lavage samples collected from the same mouse at multiple time points. (PDF) [file ppat.1012472.s001.pdf]

S1 Fig. Quantification of MmuPV1 copy number in lavages from mice infected with MmuPV1 in reproductive tract.

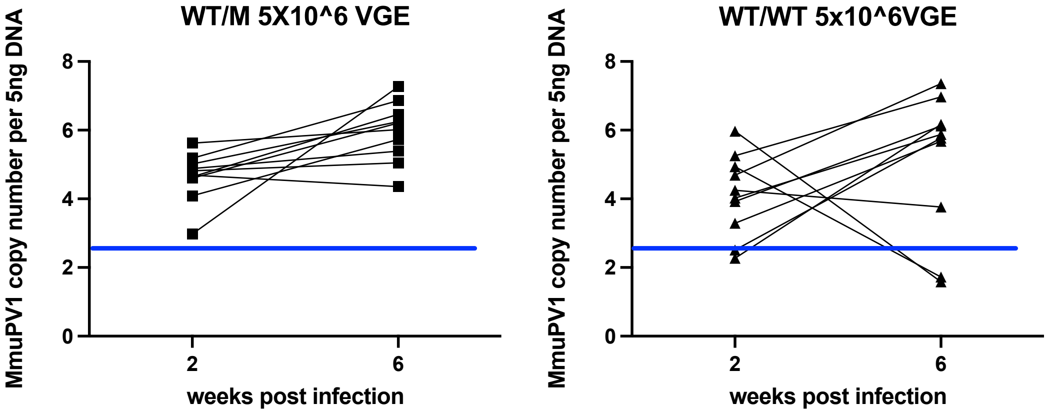

S1 Fig. Quantification of MmuPV1 copy number in lavages from mice infected with MmuPV1 in reproductive tract. Female reproductive tracts of WHIM mice (WT/M) and wildtype littermates (WT/WT) were infected with 5x10^6 VGE MmuPV1. Lavage samples were collected at 2 weeks and 6 weeks post infection. Blue line indicated MmuPV1 copy number in lavage samples from historically mock-infected control mice (background level of MmuPV1 DNA qPCR). Each line represents lavage samples collected from the same mouse at multiple time points.
