# Supplementary material for: Increased Susceptibility of WHIM Mice to Papillomavirus-induced Disease is Dependent upon Immune Cell Dysfunction
Source: PLoS Pathog. 2024 Sep 3;20(9):e1012472. doi: 10.1371/journal.ppat.1012472 (PMC11398641; doi:10.1371/journal.ppat.1012472)
Supplement: S2 Fig — Ten FFPE slides (10 micron thick) from each ear were used for DNA and RNA extraction respectively. Quantitative PCR of MmuPV1 E2 was used to quantify MmuPV1 DNA level and quantitative reverse transcription PCR of MmuPV1 E1^E4 was used to quantify MmuPV1 RNA level. Mouse GAPDH transcripts was used as house keeping gene for normalization. The difference between each group was not significant by student t-test. (PDF) [file ppat.1012472.s002.pdf]

S2 Fig. Quantification of MmuPV1 DNA copy number and normalized RNA transcripts from whole tissue.

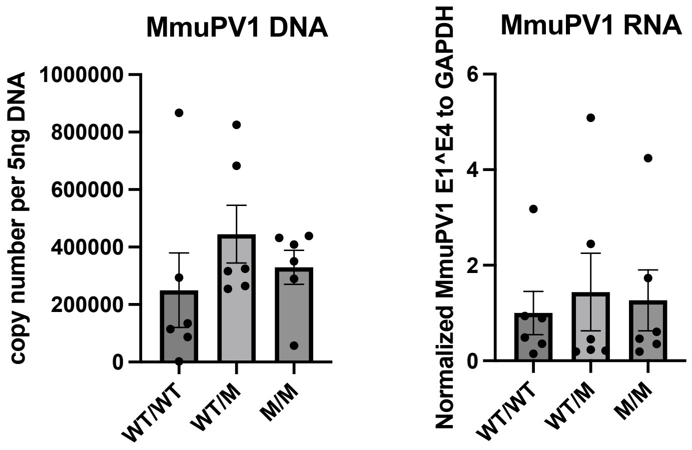

S2 Fig. Quantification of MmuPV1 DNA copy number and normalized RNA transcripts from whole tissue. Ten FFPE slides (10 micron thick) from each ear were used for DNA and RNA extraction respectively. Quantitative PCR of MmuPV1 E2 was used to quantify MmuPV1 DNA level and quantitative reverse transcription PCR of MmuPV1 E1^E4 was used to quantify MmuPV1 RNA level. Mouse GAPDH transcripts was used as house keeping gene for normalization. The difference between each group was not significant by student t-test.
