# Supplementary material for: Increased Susceptibility of WHIM Mice to Papillomavirus-induced Disease is Dependent upon Immune Cell Dysfunction
Source: PLoS Pathog. 2024 Sep 3;20(9):e1012472. doi: 10.1371/journal.ppat.1012472 (PMC11398641; doi:10.1371/journal.ppat.1012472)
Supplement: S3 Fig — WHIM mouse (M/M, CD45.1) received lethal dose 10Gy of total body irradiation. On the same day, the recipient received 8 million total bone marrow cells from a congenic wildtype donor (WT/WT, CD45.2). Blood was collected at 2 weeks, 4 weeks and 7 weeks post bone marrow transplant and were subject to flow cytometry analysis and complete blood count. A) Donor chimerism in circulating blood. B) Gating strategy for flow cytometry analysis. C) White blood cell count (WBC) and subtypes of immune cells in circulating blood of recipient mouse. Dotted lines represent average circulating count from naïve wildtype (WT) animals and WHIM heterozygous (WHIM) animals. (PDF) [file ppat.1012472.s003.pdf]

S3 Fig. Timeline establishment of donor bone marrow reconstitution in FVB/N mice.

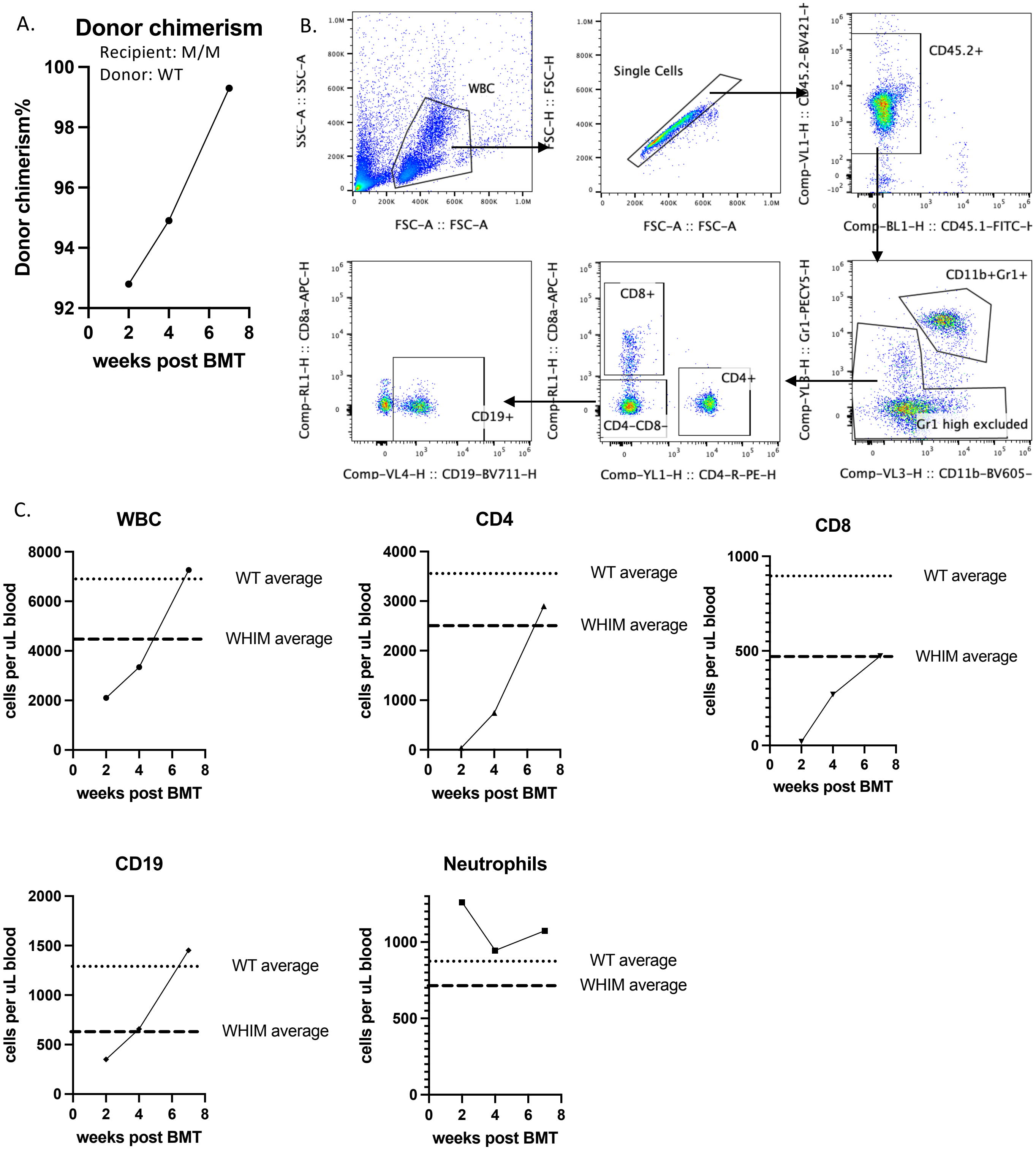

S3 Fig. Timeline establishment of donor bone marrow reconstitution in FVB/N mice. WHIM mouse (M/M, CD45.1) received lethal dose 10Gy of total body irradiation. On the same day, the recipient received 8 million total bone marrow cells from a congenic wildtype donor (WT/WT, CD45.2). Blood was collected at 2 weeks, 4 weeks and 7 weeks post bone marrow transplant and were subject to flow cytometry analysis and complete blood count. A) Donor chimerism in circulating blood. B) Gating strategy for flow cytometry analysis. C) White blood cell count (WBC) and subtypes of immune cells in circulating blood of recipient mouse. Dotted lines represent average circulating count from naïve wildtype (WT) animals and WHIM heterozygous (WHIM) animals.
