# Supplementary material for: Increased Susceptibility of WHIM Mice to Papillomavirus-induced Disease is Dependent upon Immune Cell Dysfunction
Source: PLoS Pathog. 2024 Sep 3;20(9):e1012472. doi: 10.1371/journal.ppat.1012472 (PMC11398641; doi:10.1371/journal.ppat.1012472)
Supplement: S4 Fig — Wildtype recipient mice (WT, CD45.2) received lethal dose 10Gy of total body irradiation. On the same day, the recipients received 8 million total bone marrow cells from a congenic wildtype donor (WT, CD45.1) or a heterozygous WHIM donor mouse (+/-, CD45.1). Blood was collected from a subset of recipients prior to bone marrow transplant (pre-BMT), from all recipient 7 weeks post bone marrow transplant but prior to infection (Post-BMT/Pre-infection), from all recipients 4 weeks post infection (Post-infection) to check donor reconstitution (percentage of CD45.1+ cells) in circulating blood. (PDF) [file ppat.1012472.s004.pdf]

S4 Fig. Donor chimerism in recipients.

		Pre-BMT		Post-BMT/Pre-infection		Post-infection	
recipient WHIM genotype	donor WHIM genotype	CD45.1	CD45.2	CD45.1	CD45.2	CD45.1	CD45.2
WT (CD45.2)	WT			93.20%	4.83%	92.80%	3.42%
WT (CD45.2)	WT			94.90%	3.48%	94.40%	2.69%
WT (CD45.2)	+/-			85.10%	11.60%	85.90%	7.98%
WT (CD45.2)	+/-			82.80%	13.00%	80.50%	10.60%
WT (CD45.2)	WT			85.70%	12.50%	90.30%	7.60%
WT (CD45.2)	WT			95.70%	2.47%	96.00%	1.82%
WT (CD45.2)	+/-			88.30%	8.69%	89.60%	6.00%
WT (CD45.2)	+/-			90.20%	4.96%	90.10%	4.04%
WT (CD45.2)	+/-			88.80%	7.92%	88.30%	4.23%
WT (CD45.2)	WT	0.35%	98.90%	93.60%	4.47%	95.10%	3.24%
WT (CD45.2)	+/-	7.24E-05	99.30%	83.70%	11.30%	86.10%	8.51%
WT (CD45.2)	+/-	3.08E-05	99.40%	87.90%	7.59%	87.80%	6.52%
WT (CD45.2)	+/-	2.64E-05	99.50%	77.20%	9.63%	86.40%	6.13%

S4 Fig. Donor chimerism in recipients. Wildtype recipient mice (WT, CD45.2) received lethal dose 10Gy of total body irradiation. On the same day, the recipients received 8 million total bone marrow cells from a congenic wildtype donor (WT, CD45.1) or a heterozygous WHIM donor mouse (+/-, CD45.1). Blood was collected from a subset of recipients prior to bone marrow transplant (pre-BMT), from all recipient 7 weeks post bone marrow transplant but prior to infection (Post-BMT/Pre-infection), from all recipients 4 weeks post infection (Post-infection) to check donor reconstitution (percentage of CD45.1+ cells) in circulating blood.
