# Supplementary material for: Increased Susceptibility of WHIM Mice to Papillomavirus-induced Disease is Dependent upon Immune Cell Dysfunction
Source: PLoS Pathog. 2024 Sep 3;20(9):e1012472. doi: 10.1371/journal.ppat.1012472 (PMC11398641; doi:10.1371/journal.ppat.1012472)
Supplement: S5 Fig — Wildtype mice (WT, CD45.1) or heterozygous WHIM mice (+/-, CD45.1) received lethal dose 10Gy of total body irradiation. On the same day, the recipients received 8 million total bone marrow cells from a congenic wildtype donor (WT, CD45.2). Blood was collected from a subset of recipients prior to bone marrow transplant (pre-BMT), from all recipient 7 weeks post bone marrow transplant but prior to infection (Post-BMT/Pre-infection), from all recipients 4 weeks post infection (Post-infection) to check donor reconstitution (percentage of CD45.2+ cells) in circulating blood. (PDF) [file ppat.1012472.s005.pdf]

S5 Fig. Donor chimerism in recipients.

		Pre-BMT		Post-BMT/Pre-infection		Post-infection	
recipient WHIM genotype	donor WHIM genotype	CD45.1	CD45.2	CD45.1	CD45.2	CD45.1	CD45.2
+/+	WT (CD45.2)			0.29%	96.20%	0.10%	93.90%
+/-	WT (CD45.2)			1.00%	96.10%	0.49%	94.00%
WT	WT (CD45.2)			5.74%	92.30%	4.57%	90.70%
+/-	WT (CD45.2)			1.21%	96.20%	0.59%	92.60%
+/-	WT (CD45.2)			1.59%	94.00%	1.05%	91.90%
+/-	WT (CD45.2)			2.15%	94.00%	1.22%	93.20%
+/-	WT (CD45.2)			2.57%	94.70%	1.58%	91.60%
+/+	WT (CD45.2)	98.10%	0.64%	0.60%	97.20%	0.34%	96.40%
+/-	WT (CD45.2)	98.60%	0.51%	2.74%	94.70%	1.64%	92.70%
+/+	WT (CD45.2)	97.20%	0.83%	0.43%	97.00%	0.14%	91.20%
+/-	WT (CD45.2)	98.00%	0.52%	4.33%	93.20%	0.50%	95.10%
WT	WT (CD45.2)	98.90%	0.20%	6.28%	90.30%	3.56%	91.70%
+/-	WT (CD45.2)	98.70%	0.44%	0.99%	96.70%	3.64%	88.80%
WT	WT (CD45.2)	98.80%	0.32%	4.84%	93.40%	3.52%	90.80%
WT	WT (CD45.2)	98.80%	0.27%	5.02%	92.10%	3.03%	91.50%

S5 Fig. Donor chimerism in recipients. Wildtype mice (WT, CD45.1) or heterozygous WHIM mice (+/-, CD45.1) received lethal dose 10Gy of total body irradiation. On the same day, the recipients received 8 million total bone marrow cells from a congenic wildtype donor (WT, CD45.2). Blood was collected from a subset of recipients prior to bone marrow transplant (pre-BMT), from all recipient 7 weeks post bone marrow transplant but prior to infection (Post-BMT/Pre-infection), from all recipients 4 weeks post infection (Post-infection) to check donor reconstitution (percentage of CD45.2+ cells) in circulating blood.
