# Supplementary material for: Increased Susceptibility of WHIM Mice to Papillomavirus-induced Disease is Dependent upon Immune Cell Dysfunction
Source: PLoS Pathog. 2024 Sep 3;20(9):e1012472. doi: 10.1371/journal.ppat.1012472 (PMC11398641; doi:10.1371/journal.ppat.1012472)
Supplement: S6 Fig — Ears of WHIM mice (WT/M) and wildtype mice (WT/WT) were infected with 1x10^6 VGE MmuPV1. Ten WHIM mice were implanted subcutaneously with Plerixafor-filled minipump (10mg/kg/day) at five days post infection. Nine WHIM mice and eight wildtype mice were untreated. Blood was collected from all mice before ear infection and three weeks post infection. A) Circulating blood count pre- and 3 weeks post-infection. B) Fraction of sites with warts and no warts at 4 weeks post infection. Fisher’s exact test was used to compare frequency of wart formation between groups. C) Average wart volume at 4 weeks post infection. (PDF) [file ppat.1012472.s006.pdf]

S6 Fig. Plerixafor (ADM3100) does not reverse lymphopenia or wart size in MmuPV1-infected WHIM mice.

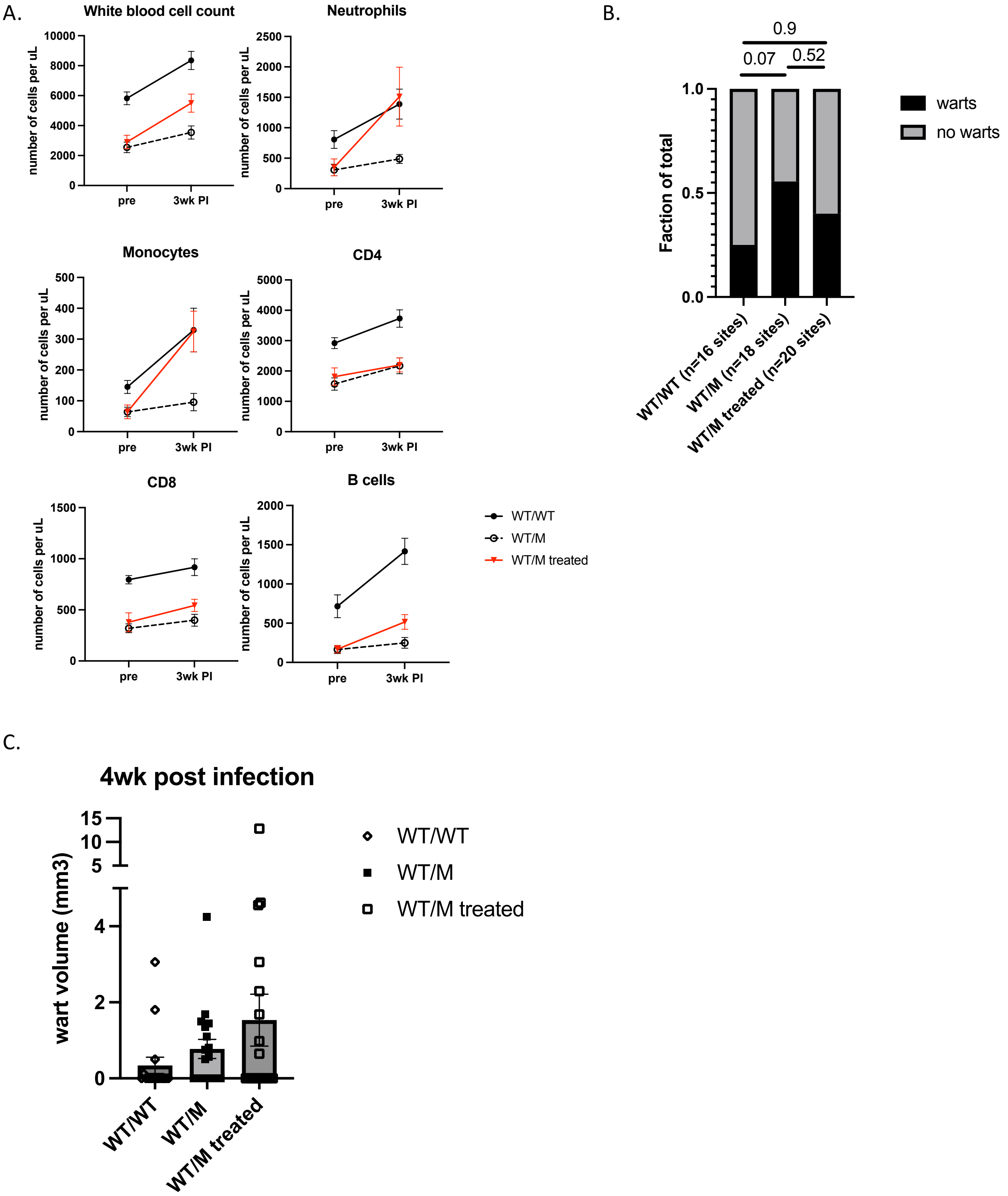

S6 Fig. Plerixafor (ADM3100) does not reverse lymphopenia or wart size in MmuPV1-infected WHIM mice. Ears of WHIM mice (WT/M) and wildtype mice (WT/WT) were infected with 1x10^6 VGE MmuPV1. Ten WHIM mice were implanted subcutaneously with Plerixafor-filled minipump (10mg/kg/day) at five days post infection. Nine WHIM mice and eight wildtype mice were untreated. Blood was collected from all mice before ear infection and three weeks post infection. A) Circulating blood count pre- and 3 weeks post-infection. B) Fraction of sites with warts and no warts at 4 weeks post infection. Fisher's exact test was used to compare frequency of wart formation between groups. C) Average wart volume at 4 weeks post infection.
